# Metrics and Algorithms for Locally Fair and Accurate Classifications using Ensembles

**DOI:** 10.1007/s13222-021-00401-y

**Published:** 2022-01-17

**Authors:** Nico Lässig, Sarah Oppold, Melanie Herschel

**Affiliations:** Institute for Parallel and Distributed Systems – Data Engineering, University of Stuttgart, Universitätsstr. 38, 70569 Stuttgart, Germany

**Keywords:** Model Fairness, Bias in Machine Learning, Model Ensembles

## Abstract

To obtain accurate predictions of classifiers, model ensembles comprising multiple trained machine learning models are nowadays used. In particular, *dynamic model ensembles* pick the most accurate model for each query object, by applying the model that performed best on similar data. Dynamic model ensembles may however suffer, similarly to single machine learning models, from bias, which can eventually lead to unfair treatment of certain groups of a general population. To mitigate unfair classification, recent work has thus proposed *fair model ensembles*, that instead of focusing (solely) on accuracy also optimize *global fairness*. While such global fairness globally minimizes bias, imbalances may persist in different regions of the data, e.g., caused by some local bias maxima leading to *local unfairness*.

Therefore, we extend our previous work by including a framework that bridges the gap between dynamic model ensembles and fair model ensembles. More precisely, we investigate the problem of devising locally fair and accurate dynamic model ensembles, which ultimately optimize for equal opportunity of similar subjects. We propose a general framework to perform this task and present several algorithms implementing the framework components. In this paper we also present a runtime-efficient framework adaptation that keeps the quality of the results on a similar level. Furthermore, new fairness metrics are presented as well as detailed informations about necessary data preparations.

Our evaluation of the framework implementations and metrics shows that our approach outperforms the state-of-the art for different types and degrees of bias present in training data in terms of both local and global fairness, while reaching comparable accuracy.

## Introduction

In decision support systems (DSS), machine learning models are frequently used to make recommendations or even decisions. While these unquestionably simplify many processes and tasks arising in modern life, critical situations emerge in automatic classification scenarios such as credit scoring, or predictive policing applications. There, critical DSS automatically assign people to classes that have the possibility to deeply affect their lives in a positive or negative way. Recent real-life examples where the use of such DSS had to be revoked due to underlying biased classifiers include an algorithm that determined A‑Level grades of British students who were unable to take their exams due to COVID-19 regulations [4]. Based on the teachers’ assessment of the student’s performance and the school’s performance in subjects, each student was assigned A‑Level grades. Using these features, about 40% of British students received lower grades than recommended by their teachers, as the model indirectly favored students from private schools and wealthy areas. After a public outcry, the algorithmic decisions were revoked and replaced by the teachers’ assessments. Another example is a recruitment tool developed by Amazon [6]. The tool was supposed to automatically assign scores to job applicants based on their application to support making hiring decisions. However, it exhibited discrimination against women, a problem that could not be resolved, leading to the project being discarded after several years of investment.

**Fig. 1:**
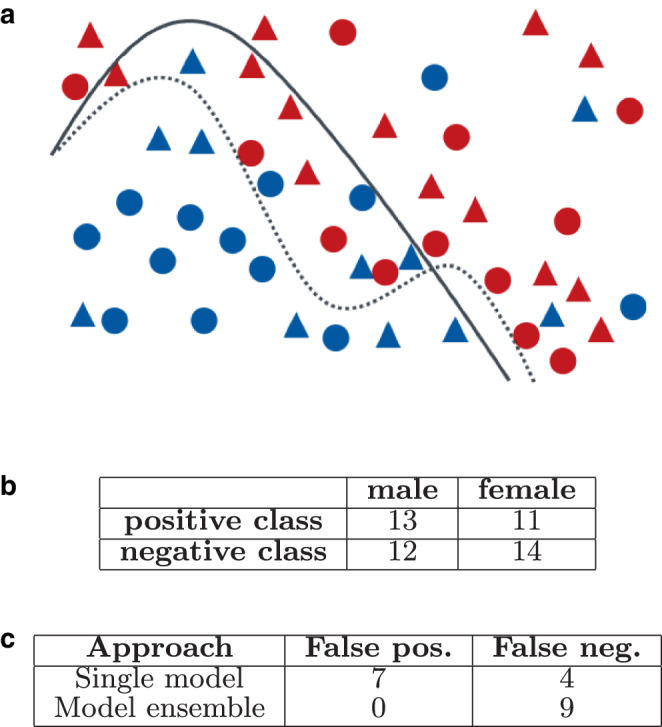
Example binary classification scenario deciding about employee raises. **a** Decision boundary for a classifier using a single model (*solid line*) and a model ensemble (*dotted line*), **b** training data statistics, **c** model performance statistics. Accuracy of single model reaches 0.78, model ensemble reaches 0.82

Classification tasks performed by DSS are, by themselves, not trivial to solve. For instance, consider Fig. 1, which summarizes a simple classification problem when deciding on employee salary raises. We visualize the training data in Fig. 1a, where we place similar employees close to each other and use different shapes to distinguish male (circle) and female (triangle) employees. The goal of the trained classifier is to divide in two classes, which we distinguish by color: employees in blue have a positive outcome and get a raise, while employees labeled in red are associated to the negative class (no raise). Opting for a simple classifier, let us assume we obtain the decision border shown as solid black line in Fig. 1a. It classifies all employees below the line into category “blue” and all persons above the line into category “red”. Using this simple classifier, a number of people are assigned to the wrong class (see Fig. 1c). We see that the simple classifier yields an accuracy of 0.78, computed as the fraction of correctly classified points vs all data points. To obtain a classifier that more faithfully reflects the complex decision boundary in our example and thus improves accuracy, we can resort to *model ensembles*. There, different (simple) models are trained and then combined, e.g., to reach a classifier with the decision border shown as a dashed line in Fig. 1a. This allows us to improve the accuracy from 0.78 to 0.82 in our example.

While the above example illustrates one means to boost the accuracy of classifiers, it leaves aside any consideration of *fairness*. The term fairness is often used in the literature to refer to non-discrimination. In the introductory examples, we see that not all students or job applicants were treated equally and some discrimination was unintentionally introduced to the classifiers. Such unfair behavior is commonly linked to some *bias*. There are many different kinds of bias that can be introduced through the data or human decisions. For instance, while it may seem reasonable to consider student’s past performance as a feature, e.g., on mock-exams to assign a final grade, wealthy students who benefit from regular tutoring may be at an advantage over students that learn for exams on their own. In case of automatic resume analysis, having a training dataset with CVs predominantly from male applicants possibly causes models that favor terms more commonly used by men than women while penalizing terms associated to women.

**Fig. 2:**
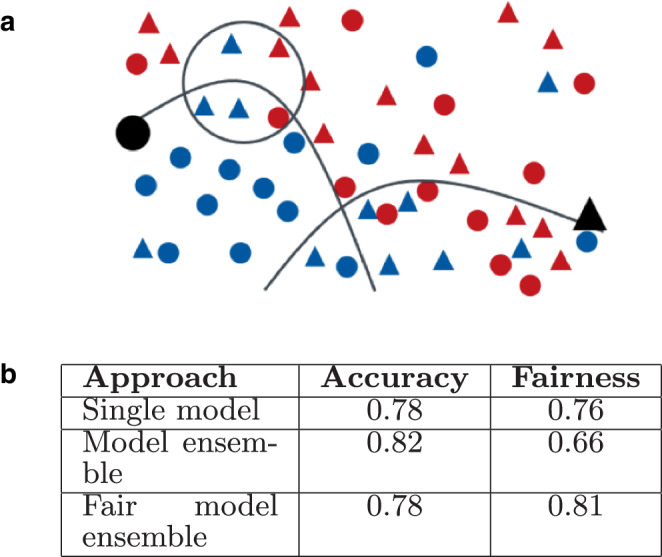
Example binary classification scenario deciding about employee raises (Fig. 1 continued). **a** Decision boundary for a fair model ensemble that combines classifiers for male (*left*) and female employees (*right*) and illustration of a locally unfair situation (circle), **b** model performance statistics

Returning to our fictional example, based on the numbers reported in Fig. 1b, we see that the training data are reasonably balanced in terms of men and women being assigned a positive or negative label, which is a good starting point. To assess the classifiers in terms of fairness, we can use one of many available bias metrics. The American Title VII of the Civil Rights Act of 1964 prohibits employment discrimination and, for example, states that there is discrimination when the probability of a woman getting a positive outcome divided by the probability of a man getting a positive outcome is less than 0.8. In the case of the single classifier and model ensemble, the value is 0.76 and 0.66 respectively (see Fig. 2b) and thus below the threshold. So using these classifiers would be against the law in the US.

With metrics quantifying bias being available, recent approaches have considered these to prevent bias. In particular, Dwork et al. [9] have introduced *fair model ensembles*. Given a pre-defined set of sensitive groups (e.g., women), their approach learns classifiers dedicated to these groups and then calculates the best combination of classifiers according to a metric that combines both fairness and accuracy. By training classifiers specialized to different groups, the approach can better capture different patterns exhibited by different groups. Optimizing for both fairness and accuracy compromises between fair treatment of the different groups and model performance. Fig. 2 illustrates the effect of using the method for fair model ensembles in our example. It combines two classifiers, for which we show the decision borders: one trained for male employees (left hand side) and one for women (right hand side). Instead of a fairness of 0.66, the positive classification of negatively labeled women by the dedicated classifier raises fairness to 0.81 and therefore (at least according to American law) no longer exhibits discrimination.

While the approach illustrated above comes closer to the goal of treating members of different predefined groups (e.g., male, female) equally, it does so from a global perspective. Thus, localized inequalities remain. For instance, looking again at Fig. 2a, while the goal of non-discrimination between women and men is met, the subregion within the depicted circle exhibits local unfairness. As a reminder, we have placed persons in the 2‑dimensional representation close to each other when they have similar features, e.g., all persons in the circled region may have similar age and number of sick days. Clearly, despite similar features, women in this region are significantly less likely to get a raise than men. This corresponds to a *local bias*. The approach presented in this paper aims at solving this issue.

The fact that optimization goals are only fulfilled globally and not locally is not a peculiarity of fairness. *Dynamic model ensembles* tackle this problem when optimizing accuracy. Intuitively, a model or model ensemble is dynamically selected for each new decision based on model performance in similar situations. This paper uses a similar rationale to optimize the overall local fairness of a model ensemble by combining ideas of both fair model ensembles and dynamic model ensembles. The contributions presented in this paper can be summarized as follows:

- We present a framework addressing the novel problem of mitigating locally unfair decisions. In an offline phase, it trains a diverse set of models to get accurate and fair models for different groups. In an online phase, it dynamically selects the model ensemble best suited for the different groups when focusing on group members similar to the subject to be classified. This combines ideas previously devised for (static) fair model ensembles and dynamic model ensembles specialized on accuracy. We also provide a runtime-efficient framework adaptation which attempts to reduce the number of computation needed for the online phase.
- We present FALCES(standing for Fair and Accurate Local Classifications using EnsembleS), which implements our framework using several algorithms. It comes in different variants, depending on whether the training data are further split before training classifiers or if trained classifiers are further pruned based on an initial assessment of their global combined performance in terms of fairness and accuracy. We now also present runtime optimized implementations based on our runtime-efficient framework adaptation.
- We propose a metric that quantifies, in a combined way, both fairness and accuracy. We study three different variants that differ in how fairness is quantified.
- We implement our algorithm variants and evaluate them on both synthetic and real data. Our results show that while we cannot fully eliminate bias, FALCES typically outperforms the state-of-the-art in both global group fairness and local group fairness, the latter quantified using a newly defined metric for local fairness. At the same time, our solution does not jeopardize accuracy. The implementations of the new runtime-efficient framework adaptation show they improve the runtime, while keeping the quality of the results on a similar level as algorithm variants implementing the original framework. Furthermore, some limits of our approach in terms of usability on different types of datasets have been found and discussed in this paper.

Note that this paper extends a previously published conference paper [14]. In particular, the new contributions of this paper include that we (i) consider alternative metrics for the accuracy and fairness measurement, (ii) devise runtime optimizations, (iii) provide some additional background on data preparation, and (iv) extend our evaluation by studying the influence of parameters and incorporating the new alternative metrics and the runtime optimized algorithm.

The remainder of this paper is structured as follows. Sect. 2 reviews related work. We present our framework in Sect. 3 and discuss algorithms implementing the framework in Sect. 4. The new runtime-efficient framework adaptation is explained in Sect. 5. Our implementation and experimental evaluation are presented in Sect. 6. The paper concludes with a summary and outlook on future research in Sect. 7.

## Related work

Our proposed solution builds on previous work on model ensembles and fairness in machine learning, in particular fair model ensembles and dynamic model ensembles.

### Model ensembles

The idea of model ensembles is to train multiple models and select or combine the best of these models [16]. Hereby, the optimization goal typically is the improvement of the accuracy of predictions [8, 17, 20]. Combining the outputs of several models has proven to be preferable compared to single-model systems. By combining the results of several models, model ensembles can, for example, reduce the risk of choosing a model that performs poorly, which reduces the overall risk of a bad decision, or overcome complex decision borders that may not be able to be implemented by a chosen model because they lie outside the functional space of the model. The same rationale underlies fair model ensembles (discussed further below), which set an additional optimization focus on increasing fairness.

### Fairness in machine learning

As already described in the introduction, the term fairness in machine learning commonly refers to the fact that models must not discriminate against people because of bias(es). Based on various laws, social definitions and understood meanings, different measures to quantify fairness have been defined [13, 15, 21]. They can be broadly classified into two categories. A group of metrics for *individual fairness* (or equality or equality of treatment) focuses on providing equal treatment to equal people [10]. However, we will focus on the second notion of fairness: *group fairness*. It is also known as equality of outcome or equity. Here, groups with different preconditions are treated differently, so that in the end everyone, despite their differences, has the same opportunities. This is intended to overcome social inequalities and offer equal opportunity to different groups [10].

Based on these fairness metrics, methods have been developed which allow the development of individual fair models using fair data, new machine learning algorithms, or techniques for modifying existing models [11, 13, 15].

### Fair model ensembles

While there is now a visible body of research on measuring fairness and learning single fair models, only few works leverage multiple models in order to achieve fairness, thereby bringing the advantages of using model ensembles to the the realm of improving fairness.

Calders and Verwer [3] create fair naive Bayes model ensembles. To this end, they split the dataset according to the favored and discriminated groups and learn a naive Bayes model on each subset with the intention to classify independently of a given sensitive attribute. An overall naive Bayes model chooses the decision of either model depending on the group of incoming data tuples to be classified. While this approach yields fairer models, it is specialized to and does not extend beyond naive Bayes models.

Dwork et al. [9] combine multiple machine learning classifiers to improve group fairness. They provide different versions of their algorithm, where the models are either learned on the different subgroups as in [3] or on larger data subsets in order to prevent accuracy loss. Their approach uses a joint loss metric that optimizes for both accuracy and fairness in order to assess which model should be used for which group of the dataset. While this approach does consider both accuracy and fairness at group level using any type of classifier, it may suffer from local unfairness.

### Dynamic model ensembles

Dynamic classifier selection [5] selects one classifier during runtime for each new sample which has to be classified. This is based on the rationale of model ensembles that not every classifier is an expert in all local regions of the feature space. Usually, for each new sample the local region is first identified, for example using $k$-nearest-neighbors (kNN). Then, the quality of available classifiers is determined and the best one(s) are selected. Dynamic ensemble selection is similar, it merely selects ensembles instead of classifiers. One example is the Dynamic Classifier Selection by Local Accuracy (DCS-LA) algorithm by Woods et al. [19]. First, it uses kNN to identify the local region. Then, local accuracy of classifiers is evaluated as percentage of correctly classified samples in the local region. Alternatively, it uses local class accuracy, which is the accuracy of classifiers in the local regions with respect to the class the classifiers assign to the new sample. Only the most accurate classifier is then used to classify the unknown sample.

## Framework for fair and dynamic model ensembles

As motivated in the introduction, our goal is to combine the benefits of fair model ensembles on the one hand and dynamic model ensembles on the other hand to devise a solution that resolves not only global bias among different groups, but also local bias, while not compromising overall accuracy. The rationale is that, while it is typically possible to define groups that should be treated fairly (and that are often defined by law), it is quite challenging to fully anticipate variations (sub-groups) within these groups that indirectly cause local bias. Techniques to counter local bias can potentially help in reaching equal opportunity among groups with similar features or similar trajectory. In this section, we first formalize our problem statement to counter locally unfair decisions. We then present a framework where we combine the ideas of fair and dynamic model ensembles to solve this problem.

**Fig. 3:**
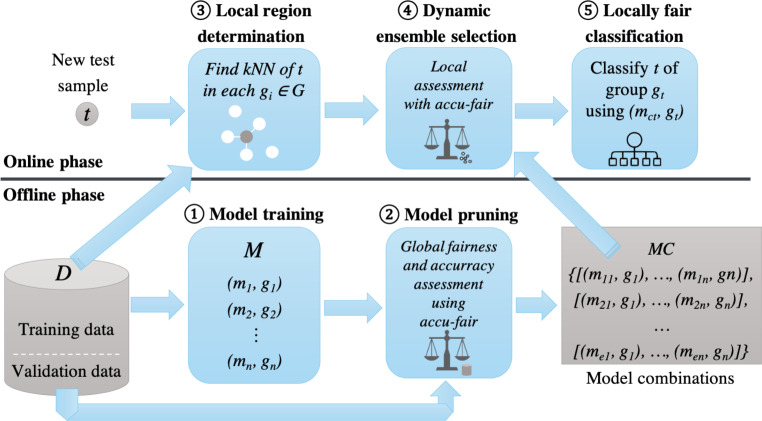
Framework for locally fair classifications by combining fair and dynamic model ensembles

### Locally unfair decisions

As illustrated in Fig. 2a, the problem with locally unjust decisions is that while existing solutions (reviewed in Sect. 2) are optimized to make globally fair and accurate decisions, there are still local regions where data points of different groups are treated unfairly. To address this problem, the decision should be optimized so that the optimal (i.e. fair and accurate) decision can be made at a local level. Our emphasis in this paper is on *group fairness*, i.e., equal opportunities between groups.

Formally, we consider as given a labeled dataset $D$, a similarity metric $s$, a positive integer $k$ indicating a local region sample size, an optimization metric $af$ combining fairness and accuracy, and a set of machine learning models (classifiers) $M$ for the same classification task. Furthermore, $D$ can be partitioned into groups $G$, for which equal opportunity is relevant. We further assume a new test sample $t$ that belongs to one of the groups $G$. Then, we define our goal of *locally fair and accurate classification* as a classification task that classifies $t$ using a model $m\in M$ with the best performance according to the $af$ metric in the local region of $D$ around $t$. This local region includes the $k$ items in $D$ most similar to $t$ according to $s$.

### Framework

To address the problem defined above, we combine the rationales underlying both fair and dynamic model ensembles described in Sect. 2 into a new framework for fair and dynamic model ensembles. The main components of this framework are visualized in Fig. 3. We distinguish between an *offline phase* (bottom part), where suited model ensembles are trained and selected, and an *online phase* (upper part), where a previously unseen test sample $t$ is classified by dynamically selecting the model ensemble most appropriate for $t$.

**Offline phase.** The first step of the offline phase, named *model training*, is a step common to model ensemble approaches. Here, using training data taken from the labeled dataset $D$, a diverse set of classifiers is trained. Given that we target both fair and accurate decisions, model training can benefit from using different subsets of data based on the different groups $G$ present in $D$, which has for instance been proposed for fair model ensembles (see Sect. 2). We denote the set of classifiers resulting from model training considering different groups $G$ as $M=\{(m_{1},g_{1}),(m_{2},g_{2}),\ldots,(m_{n},g_{n})\}$, where each $m_{i}$ is a model and $g_{i}$ identifies the group it is trained for. Among these classifiers, not all may be suited to make both fair and accurate decisions. Also, too many classifiers to be considered during the online phase (further discussed below) can be computationally prohibitive. Therefore, during *model pruning*, the framework assesses all model combinations or ensembles possible with the classifiers in $M$ that use exactly one classifier per group $g_{i}$. Assessment is done with respect to the global performance metric $af$ that considers both accuracy and fairness. Only the best classifier combinations are retained after model pruning, resulting in the model candidates *MC* = {[((*m*_11_, *g*_1_) …, (*m*_1*n*_, *g*_*n*_)], …, [((*m*_*e*1_, *g*_1_) …, (*m*_*en*_, *g*_*n*_)]}, a set of model ensembles (in square brackets) s.t. for each ensemble, $g_{i}\neq g_{j}$ when $i\neq j$ and $n=|G|$.

**Online phase.** When a new test sample $t$ is to be classified, the framework determines the local region $t$ belongs to as part of *local region determination*. To this end, it performs a kNN search of $t$ on each $g_{i}$, where $g_{i}$ is a group in $G=\{g_{i},\ldots,g_{n}\}$. The framework specifically selects an equal number of members similar to $t$ of each group, to have a locally balanced data region with respect to the different groups. Then, for this particular region, *dynamic ensemble selection* assesses which ensemble $E\in MC$ achieves the best local performance with respect to $af$. Intuitively, this dynamically selects the optimal model ensemble comprising a dedicated model for each group for the region most relevant to $t$. With this approach, our framework combines previous techniques separately devised for fair and dynamic model ensembles. The identification of the locally best model is performed according to dynamic model ensemble techniques using fair model ensemble metrics. Therefore the classifiers are tested on the local region of the test sample using an $af$ metric. Finally, the best classifier $m_{ct}$ such that $(m_{ct},g_{t})\in E$ and $g_{t}$ corresponds to the group $t$ belongs to is used in the final step of *locally fair classification* to classify $t$.

## Algorithms implementing the framework

Sect. 3 discussed the general framework that we propose for locally fair and accurate classifications. There are a variety of techniques from both fair and dynamic model ensemble research, which can be applied or extended to implement its components. In the following, we discuss the algorithms we consider to implement the framework that stand behind our FALCES system.

### Model training

As mentioned before, the set of classifiers should be diverse in order to benefit from combining them to model ensembles. To this end, we vary both the set of machine learning techniques used to train classifiers as well as the data from $D$ that are considered for training.

In principle, any machine learning technique suited for classification tasks can be considered as a candidate technique. In our evaluation, we will resort to simple techniques, e.g., decision trees, logistic regression, or nonlinear support vector machines [12].

Concerning the data, following previous research on fair model ensembles [3, 9], we consider splitting the input dataset $D$ on pre-defined sub-datasets that correspond to the different groups for which we aim to achieve group fairness (e.g., we divide by sex (male, female) and race (white, others) in our experiments which creates four subgroups). This effectively partitions $D$ into $G=\{g_{1},\ldots,g_{n}\}$, assuming $n$ distinct groups. Then, models are trained separately for the different partitions. Different training datasets have the advantage of learning different models that exhibit their strengths in certain areas of the feature space. However, as shown in Calders et al [3], splitting up the input dataset does not necessarily improve the quality of the results. In addition, complex decision borders between the two groups, which originate from different behavioral patterns, can be better modeled, thus increasing accuracy [9].

As a result, similar to [3, 9], we obtain classifiers that are “specialized” on some group. More precisely, in this variant, we obtain $M=\{(m_{1},g_{1}),\ldots,(m_{k},g_{n})\}$ where each $(m_{i},g_{j})$ associates a classifier $m_{i}$ to a group $g_{j}$. For any two $(m_{i},g_{j}),(m_{i^{{}^{\prime}}},g_{j^{{}^{\prime}}})$, it holds that $m_{i}\neq m_{i^{{}^{\prime}}}$, and $g_{j},g_{j^{{}^{\prime}}}\in G$, but it is possible that $g_{j}=g_{j^{{}^{\prime}}}$.

#### *Example 1 *

As a simple example, consider we spilt a sample dataset following the gender of employees, which results in a group for each gender, e.g., $g_{F}$ for female employees and $g_{M}$ for male employees. Assuming $m_{1},m_{2},m_{3}$ are trained on $g_{M}$ and $m_{4},m_{5},m_{6}$ on $g_{F}$, we obtain *M* = {(*m*_1_, *g*_*M*_), (*m*_2_, *g*_*M*_), (*m*_3_, *g*_*M*_), (*m*_4_, *g*_*F*_), (*m*_5_, *g*_*F*_), (*m*_6_, *g*_*F*_)}.

On the downside, splitting the data as described above can lead to a too small dataset to train on, which often results in loss of accuracy for the classifiers. Hence, we further consider the option of training models on the complete dataset $D$ rather than on individual partitions of $G$. In this setting, we have $M=\{(m_{1},g_{D}),\ldots(m_{n},g_{D})\}$, where $g_{D}=D$.

To distinguish the two variants for implementing model training described above in FALCES, we will append a suffix SBT (for Split Before Training) for the first option, while absence of this suffix indicates training is performed on the full training dataset.

### Model pruning

In the offline phase, the number of classifiers can already be reduced based on their global performance in order to improve the efficiency of the online phase later. Indeed, the less classifiers need to be considered in the online phase, the faster the classification of a new test item is. As we shall see in the evaluation (Sect. 6.8), this has only little impact on making locally fair and accurate decisions, while runtime may improve significantly. In this section, we first present metrics we consider for model pruning, to then describe how these are used for model pruning.

#### Metrics

To assess the performance of a model when considering both accuracy and fairness, we rely on a metric that combines these two dimensions, denoted as $af$. To the best of our knowledge, the state-of-the art metric that accounts for both accuracy and fairness is the metric proposed by Dwork et al. [9] for fair model ensembles, defined as follows.

1 $$\hat{L}=\underbrace{\frac{\lambda}{|D|}\sum\limits_{t_{i}\in D}|y_{i}-z_{i}|}_{\text{Inaccuracy}}+\underbrace{\frac{1-\lambda}{|D|}\sum\limits_{g_{j}\in G}\left|\sum\limits_{t_{i}\in D:g_{t_{i}}=g_{j}}{z_{i}}-\frac{1}{|G|}\sum\limits_{t_{i}\in D}z_{i}\right|}_{\text{Unfairness}}$$

Here, the number of tuples in a labeled dataset $D$ is denoted as $|D|$, each tuple $t_{i}\in D$ has an actual and predicted label denoted as $y_{i}$ and $z_{i}$ respectively, $|G|$ represents the number of different groups in $G$, $g_{t_{i}}\in G$ represents the group a tuple $t_{i}$ belongs to, and $0\leq\lambda\leq 1$ balances the relative weight of the accuracy and the fairness part of the equation. Intuitively, in the first part of the metric, accuracy is measured by comparing the predicted and actual label for each data tuple (also known as $L_{1}$ loss). The second part of the metric determines the fairness of the classifier combination that associates a classifier to each group. It sums up the difference between the sum of predicted values of each group and the overall sum of labels divided by number of groups. Note that for both sides, higher values actually mean a poorer performance, we thus qualify them as inaccuracy and unfairness.

This metric combines both accuracy and fairness, however, the fairness-part is sensitive to differences in the relative size of groups. Assume for instance there is a larger group $g_{L}$ and a smaller group $g_{S}$ with equal sum of $z_{i}$, i.e. equal number of positively classified tuples. Indeed, the metric considers the situation to be fair among these two groups (unfairness-part drops to 0), even though the probability that a member of $g_{L}$ is assigned a positive label is smaller than the probability of a member of $g_{S}$ being assigned a positive label. While this may well serve minorities that are considered protected groups and are thus indirectly favored by being part of the smaller group, it does not accurately reflect equal opportunity.

Therefore, we introduce three alternatives to Eq. 1, that address this shortcoming. They all differ in the fairness part of the equation. Eq. 2 is based on mean-difference, Eqs. 3 and 4 are adaptations of the impact ratio and elift ratio [21] respectively and substitutes for the fairness part of Eq. 2.

**Unfairness based on mean-difference.** Our first metric for $af$ modifies the fairness-related part of Eq. 1 to also consider the number of tuples $|g_{j}|$ in a group $g_{j}\in G$. This results in the following metric.

2 $$\hat{L}_{\text{mean}}={\frac{\lambda}{|D|}\sum\limits_{t_{i}\in D}|y_{i}-z_{i}|}+{\frac{1-\lambda}{|G|}\sum\limits_{g_{j}\in G}\left|\sum\limits_{t_{i}\in D:g_{t_{i}}=g_{j}}\frac{z_{i}}{|g_{j}|}-\frac{1}{|D|}\sum\limits_{t_{i}\in D}z_{i}\right|}$$

While the accuracy part still determines $L_{1}$ loss, the fairness part has slightly changed. For each group, its mean value is set in relation to its group size and compared to the overall mean value of positive predicted labels. These are then again summed up and divided by the number of groups to allow for an arbitrary number of groups.

**Unfairness based on impact ratio.** The impact ratio metric [21] measures the ratio of the probabilities of positively predicted labels of an unfavored group to the favored group. An impact ratio of 0 indicates unfairness, while an impact ratio of 1 indicates complete fairness. In this metric, the result can exceed 1, if the unfavored group has a higher chance of a positive outcome than the favored group. To ensure the range of the impact ratio, we inverse the fraction when the unfavored group has favored values. We do this as we aim to achieve total equality among all groups. Hence, to retain a quantification of unfairness over all described $af$ metrics, we subtract the impact ratio value, ensured to be between 0 and 1, from 1. Thus, the impact ratio adaptation denoted as $\text{impact}^{*}$ is formally defined as follows. Here, $g_{0}$ denotes the favored group.

3 $$\text{impact}^{*}=\begin{cases}1-\dfrac{\sum\limits_{t_{i}\in D:g_{t_{i}}=g_{j}}\frac{z_{i}}{|g_{j}|}}{\sum\limits_{t_{i}\in D:g_{t_{i}}=g_{0}}\frac{z_{i}}{|g_{0}|}},&\text{if}\leavevmode\nobreak\ \sum\limits_{t_{i}\in D:g_{t_{i}}=g_{j}}\frac{z_{i}}{|g_{j}|}<\sum\limits_{t_{i}\in D:g_{t_{i}}=g_{0}}\frac{z_{i}}{|g_{0}|}\\ 1-\dfrac{\sum\limits_{t_{i}\in D:g_{t_{i}}=g_{0}}\frac{z_{i}}{|g_{0}|}}{\sum\limits_{t_{i}\in D:g_{t_{i}}=g_{j}}\frac{z_{i}}{|g_{j}|}},&\text{if}\leavevmode\nobreak\ \sum\limits_{t_{i}\in D:g_{t_{i}}=g_{j}}\frac{z_{i}}{|g_{j}|}> \sum\limits_{t_{i}\in D:g_{t_{i}}=g_{0}}\frac{z_{i}}{|g_{0}|}\\ 0&\text{otherwise}\end{cases}$$

Similarly to the mean difference, $\text{impact}^{*}$ has to be summed up over all groups. Thus, we define $\hat{L}_{\text{impact}^{*}}$ as Eq. 2 where $\text{impact}^{*}$ substitutes the content between the absolute values bars.

**Unfairness based on elift ratio.** The elift ratio fairness metric [21] is based on the same underlying idea as the impact ratio, but instead of measuring the ratio to the favored group, it measures the ratio to the overall average probability of positive predicted labels. Analogously to the impact ratio, we “convert” it to an unfairness metric in the range $[1,0]$. We denote our adaptation as $\text{elift}^{*}$, which is defined by the following equation.

4 $$\text{elift}^{*}=\begin{cases}1-\dfrac{\sum\limits_{t_{i}\in D:g_{t_{i}}=g_{j}}\frac{z_{i}}{|g_{j}|}}{\frac{1}{|D|}\sum\limits_{t_{i}\in D}z_{i}},&\text{if}\leavevmode\nobreak\ \sum\limits_{t_{i}\in D:g_{t_{i}}=g_{j}}\frac{z_{i}}{|g_{j}|}<\frac{1}{|D|}\sum\limits_{t_{i}\in D}z_{i}\\ 1-\dfrac{\frac{1}{|D|}\sum\limits_{t_{i}\in D}z_{i}}{\sum\limits_{t_{i}\in D:g_{t_{i}}=g_{j}}\frac{z_{i}}{|g_{j}|}},&\text{if}\leavevmode\nobreak\ \sum\limits_{t_{i}\in D:g_{t_{i}}=g_{j}}\frac{z_{i}}{|g_{j}|}> \frac{1}{|D|}\sum\limits_{t_{i}\in D}z_{i}\\ 0&\text{otherwise}\end{cases}$$

Similarly to $\hat{L}_{\text{impact}^{*}}$, we define $\hat{L}_{\text{elift}^{*}}$ as Eq. 2 where $\text{elift}^{*}$ substitutes the contents between the absolute values bars.

#### Pruning procedure

Using a combined accuracy and fairness metric as defined in the previous section, model pruning aims at retaining only a “good” selection of ensembles formed by models of $M$ obtained during model training. Given that we are using model ensembles, this evaluation of model quality is performed by considering all possible combinations of classifiers in $M$ when choosing one classifier per group, and keeping only the best ones. In our implementation, we keep ensembles up to a predefined rank. Another possibility would be to use a threshold for the maximally acceptable $af$ score.

##### *Example 2 *

Continuing our previous example, given that we have three classifiers dedicated to $g_{F}$ and $g_{M}$, respectively, we have a total of 9 combinations to test using $af$. Let us assume that the top‑2 ensembles according to $af$ are $(m_{1},m_{4}),(m_{2},m_{5})$. Assuming FALCES is configured to the top‑2 combinations, we obtain $MC=\{[(m_{1},g_{M}),(m_{4},g_{F})],[(m_{2},g_{M}),(m_{5},g_{F})]\}$.

Similarly to model training, we consider running FALCES with or without model pruning enabled. When active, we append PFA to the algorithm name.

### Local region determination

Moving on to the online phase, the task is to classify a new tuple $t$ in a locally accurate and fair manner. Our framework defines locality relying on a similarity measure $s$ and considers retrieving the $k$ most similar tuples to $t$ in $D$.

The approach for retrieving the most similar tuples depends on the types of data we are dealing with. We now discuss different types of data and present the corresponding algorithms to retrieve the $k$ most similar tuples.

#### Numerical data

When a dataset containing only numerical data is used, i.e. the values of attributes are quantitatively comparable, one way to determine the $k$ most similar tuples are kNN algorithms [2]. FALCES uses the $kd$-tree nearest neighbor approach [1] because it is simple and efficient. This method creates a $k$-dimensional tree that can be precomputed during the offline phase in which the tuples from $D$ are arranged and stored according to the dimensions. During the online phase, when the tuple $t$ is to be classified, the tree can then be searched in $O(\log|D|)$ time.

While searching for the nearest neighbors, we need a similarity metric to identify tuples similar to $t$. To compare two tuples $t_{1}=(x_{1},{\ldots},x_{n})$ and $t_{2}=(y_{1},{\ldots}y_{n})$, FALCES uses the Minkowski-Distance $md(t_{1},t_{2})=$ ${\left(\sum_{i=1}^{n}{|x_{i}-y_{i}|}^{p}\right)}^{\frac{1}{p}}$. It is a generalization of both the Manhattan distance ($p=1$) and the Euclidean distance ($p=2$) and has already proven useful for similar problems such as K‑Means algorithms [18].

Using this distance measure, we identify the nearest neighbors of $t$. However, it must be ensured at this step already that the $af$ metric used in the next step of FALCES receives the necessary information to calculate group fairness. For this, it needs to receive tuples from all groups to be able to produce meaningful results. Therefore, in FALCES, the kNN algorithm is applied to each group separately, which results in $|G|$ trees and $|G|\cdot k$ nearest neighbors, where $|G|$ is the number of groups considered.

For better results, the comparable values should be within a given range. If, for example, the maximum difference of one attribute is 100 and 1 for another attribute, the first attribute would have a bigger influence on the overall result of the kNN algorithm. Therefore, normalizing the numerical data in a preprocessing step could be useful.

The runtime complexity using the kNN algorithm would be $O(\log n)$ for finding the $k$ most similar tuples of one sample, where $n$ is the number of points in the dataset. In addition to this, the construction of the $k$–$d$ tree takes $O(n(d+\log n))$, where additionally $d$ is the dimensionality of the dataset, but it only has to be constructed once per group.

#### Nominal data

Often, the datasets we receive contain important nominal attributes, i.e., non-rankable categories. In order to use the kNN algorithm, defined in Sect. 4.3.1, to retrieve the $k$ most similar tuples of a sample, the data has to be preprocessed.

If the nominal attributes are binary, this is a simple task. Both categories can be e.g. mapped to either value 0 or value 1, respectively. However, for non-binary nominal attributes this is not possible. For non-binary nominal attributes we perform a one-hot encoding. That is, we add a new attribute for each of the possible nominal values and set the value to 1 if the sample has the corresponding attribute, 0 otherwise. The drawbacks of this approach is that there has to be a set of possible nominal values and it drastically increases the number of dimensions. Especially for datasets with attributes with many different possible nominal values, the resulting preprocessed dataset would be too huge in terms of dimensionality.

Therefore, we implement an algorithm for nominal data that does not need preprocessed data as input. Given two samples, the algorithm returns 0, if their nominal attribute value is equal, else it returns 1. The algorithm has to be executed for each attribute. A low overall value between two samples indicates that they are similar. The runtime complexity of this naive approach is $O(n\cdot d)$ per prediction point, with $n$ being the number of points in the dataset and $d$ being the dimensionality of the dataset. Therefore the runtime is way higher compared to the runtime complexity of the kNN algorithm.

#### Ordinal data

Many datasets also use ordinal data, i.e. rankable categorical values. In order to apply the kNN algorithm, we again preprocess the data. Therefore, the ordinal data values are mapped to numerical values, where lower values indicate closely ranked ordinal values. Without preprocessing, the data is handled like nominal data.

**Table 1:** Example extract of an income dataset

Name	Experience	Education	…
Mark B.	13 y.	No college degree	$\cdots$
Sue C.	13 y.	MSc.	$\cdots$
Jane D.	14 y.	BSc.	$\cdots$
$\vdots$	$\vdots$	$\vdots$	$\vdots$

Preprocessing ordinal data is not only useful because the runtime-efficient kNN algorithm can be applied. It can also improve the quality of the overall results compared to the naive algorithm described in Sect. 4.3.2. We illustrate this quality effect using the example in Table 1. During local region determination, the nearest neighbor of Sue has to be found in order to predict her salary. We assume, that Mark, Sue and Jane only differ in experience and education. We could apply a naive strategy, where we consider two people more similar, if an attribute value is exactly the same. Then Sue’s next neighbor would be Mark, since they have the same experience value. However, realistically, Sue would be expected to have a closer salary to Jane, considering that their educational level is very similar and their experience is not equal, but close enough. Hence, being able to more accurately model the difference between Master of Science, Bachelor of Science and no degree is also useful in terms of result quality.

The preprocessing step of ordinal data does not effect the dimensionality of the dataset. Here each value can be mapped directly onto corresponding numerical values indicating their rank within the attribute.

### Dynamic ensemble selection

Based on the $|G|\cdot k$ tuples defining the local region for a given test sample $t$, dynamic ensemble selection identifies the ensemble $E=[(m_{c_{1}},g_{1}),\ldots,(m_{c_{p}},g_{p})]\in MC$ that achieves the best local performance. To this end, FALCES follows previous research on dynamic model ensembles [19] and combines these techniques with the $af$ metric. More precisely, using as input $MC$, we evaluate all model combinations based on $af$ when they classify the $|G|\cdot k$ nearest neighbors of $t$. The combination $E$ with the lowest $af$-score is retained.

#### *Example 3 *

Assume we want to classify a male employee $t$ that is thus considered to be part of $g_{M}$. Assuming $k=20$, kNN retrieves the 20 male and 20 female samples in $D$ most similar to $t$. The two combinations possible with the classifiers retained after model pruning (see Example 2), i.e., $[(m_{1},g_{M}),(m_{4},g_{F})]$ and $[(m_{2},g_{M}),(m_{5},g_{F})]$, are evaluated using the $af$ metric and focusing on the 40 samples of $D$ that form the local region. In our example, let this result in $E=[(m_{1},g_{M}),(m_{4},g_{F})]$ as this combination reaches the lowest score.

Note that through previous model pruning during the offline phase, the above example needed only to consider 2 instead of 25 classifier combinations. In addition to reducing the number of comparisons, we further reduce the complexity of an individual combination assessment, because the computation of classification predictions for all sets of classifiers and all local $|G|\cdot k$ tuples can be quite time consuming. That is, FALCES precomputes all classification predictions for all tuples in $D$ using all models in $M$. This allows dynamic ensemble selection to simply look up the necessary predictions instead of repeatedly computing them by applying the classifier for each test sample during the online phase.

### Locally fair classification

Finally, the classifier of the previously identified model ensemble $E$ that achieved best local performance with respect to the $af$ metric and that is associated to the same group as $t$ is used to classify $t$.

#### *Example 4 *

Continuing our running example with $E=[(m_{1},g_{M}),(m_{4},g_{F})]$, $m_{1}$ is finally used to classify $t$, because $t$ belongs to $g_{M}$. Considering a different $t^{\prime}$ of group $g_{M}$ may result in a different local region, where for instance $E=[(m_{2},g_{M}),(m_{5},g_{F})]$ performs better, resulting in the use of $m_{2}$ instead.

## Runtime-Efficient Framework Adaptation

The major runtime bottlenecks of the FALCES algorithm are the determination of local kNN and dynamic ensemble selection. We already have addressed methods to reduce the runtime of the kNN step using preprocessed data on an algorithmic level (see Sect. 4.3).

**Fig. 4:**
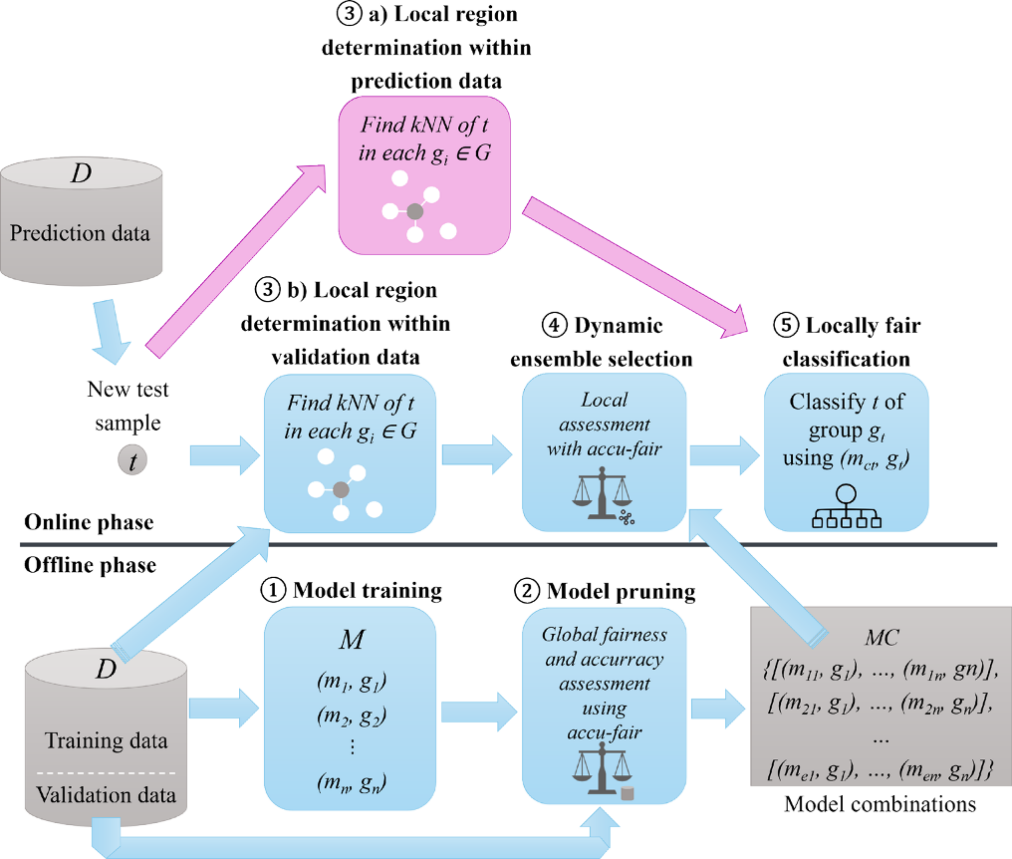
Runtime-efficient framework adaptation

Additionally, we provide an adaptation, depicted in Fig. 4, of the original framework to reduce the runtime of the online phase. The goal is to reduce the number of computations of the steps causing the runtime bottlenecks. Here the idea is to add another kNN step (3a) in which we want to find the kNN of the current, not yet predicted, test sample $t$. Step 3a) can be computed in parallel to step 3b) and step 4. After the locally best model combination is found for the current test sample, we then use this combination to classify $t$, as well as all kNN of $t$ within the prediction dataset. With this method we can reduce the amount of times step 3b) and step 4 have to be computed, since we do not have to redo them for the corresponding kNN of $t$.

This strategy is useful, when we already have a dataset of which we want to predict the label, instead of just predicting the label of one sample. The FALCES algorithms described in Sect. 4 can be adapted by adding the new step to the online phase. The runtime would reduce by the factor of the number of kNN to be considered within the prediction dataset.

Another improvement on the runtime can be done through sorting the indices within the validation dataset beforehand. This does not have an influence on the overall quality of the results, and thus can be used for both frameworks.

## Evaluation

We have implemented the algorithms discussed in Sect. 4 and present their evaluation in this section. We first describe our experimental setup. We then present and discuss results on combined accuracy and fairness, differences observed for the different metrics used, as well as differences when using different parameter settings ($\lambda$ value of the metrics and $k$-value of the kNN algorithm). Furthermore the impact of preprocessed data is observed and we compare the original algorithms with their new runtime-efficient framework adaptations defined in Sect. 5. Additionally the runtime results on the online phase are discussed as well.

### Experimental setup

This section summarizes which different algorithm variants and baseline solutions we compare in our evaluation. We further discuss datasets and metrics we use for benchmarking.

**Compared algorithms.** We have presented different variants of FALCES, depending on whether or not the training data is split before training and whether or not model pruning is applied. In addition, we compare to the state-of-the-art algorithms. More precisely, we consider the algorithms summarized in Table 2 for each experiment. Additionally, we also want to evaluate the performance of the algorithm variants using the runtime-efficient framework adaptation described in Sect. 5 and compare it with the original FALCES variants. We evaluate the different fairness metrics for these algorithms as well as vary the $\lambda$ value of the $af$ metrics used. Furthermore we want to see what influence changing the $k$-value for the kNN algorithm has on the overall results. When not mentioned otherwise, we set $k=15$ as the default value. The reasoning for choosing this value has been based on some prior testing, which showed across the datasets considered that a $k$-value of 15 provides promising results, while still having an acceptable runtime.

**Datasets and ML models.** We use both synthetic and real benchmark data in our evaluation.

**Table 2:** Overview of the algorithms compared in our evaluation

Algorithm	Description
FALCES	Our baseline algorithm without splitting before training and without model pruning.
FALCES-SBT	This variant of FALCES splits the dataset for training but does not apply model pruning.
FALCES-PFA	In this variant, model pruning is applied on models trained over the complete training dataset.
FALCES-SBT-PFA	The offline phase performs model pruning on models that have been trained on sub-sets of the training dataset, which has been split according to considered groups.
DCS-LA [19]	A baseline algorithm for dynamic model ensembles that optimizes accuracy, which we extended for FALCES.
Decouple [9]	State-of-the-art algorithm for fair model ensembles, when models are trained using the full training dataset.
Decouple-SBT[9]	Variant of Decouple that trains models on a previously split training dataset.

**Table 3:** Different configurations for generating synthetic data, default values in bold

Bias type	Parameter settings
Group balance	0.1, 0.2, 0.3, 0.4, **0.5**
Social bias	**0**, 0.1, 0.2, 0.3, 0.4, 0.5
Implicit bias	**0**, 0.1, 0.2, 0.3, 0.4, 0.5
Preprocessing	**on**, off

**Table 4:** Different parameter settings used in the evaluation, default values in bold

Parameter	Parameter settings
Metric	$\hat{\mathbf{L}}_{\textbf{mean}}$, $\hat{L}_{\text{impact}^{*}}$, $\hat{L}_{\text{elift}^{*}}$
$k$-value of kNN	5, 10, **15**, 20, 25
Framework adaptation	on, **off**

We developed a synthetic data generator in order to control different types and degrees of bias in order to study how the different algorithms are affected by these. The generated datasets use numerical values, hence the kNN algorithm can be directly used on the datasets. It generates labeled data for two groups and a binary classification task and allows to control (i) the *group balance*, (ii) the degree of *social bias*, and (iii) *implicit bias*. Group balance describes the percentage group $g_{1}$ represents in the full dataset ($g_{2}$ implicitly making up the remainder of the dataset), which can be very unbalanced (e.g., only 10% of the training data belong to $g_{1}$) or perfectly balanced at $50\%$. Social bias refers to bias directly related to the protected attribute defining a group (e.g., gender in our example), reflected by different probabilities for a positive label in the different groups (e.g., women have a lower probability for a positive label than men). Such bias is sometimes also called historical bias, because it reflects direct discrimination of a group in a dataset that commonly labels data based on historical decisions. In our experiments, a social bias of 0 means probabilities are equal for both groups (no discrimination), 0.1 if the probability for $g_{1}$ differs by 0.1, and so on. Implicit bias is present in a dataset when, even though groups are not directly discriminated, their label depends on an unfavorable attribute value that occurs more frequently in the protected group, i.e., that is correlated to the protected group. Note that both examples from the press mentioned in the introduction are likely linked to such implicit bias. Similarly to social bias, we vary implicit bias from 0 (none) in increments of 0.1. The generated data in all cases consist of approximately 13,000 tuples. Table 3 summarizes the configurations we used for testing. When not mentioned otherwise, the values are set to the default values highlighted in bold.

We chose the Adult Data Set [7], a census income dataset with data from 1994, which is a commonly used dataset in multiple machine learning experiments. This dataset consists of approximately 49,000 tuples and contains various variables, including a binary salary value of yearly income with the threshold of 50 K$, which is our label in the experiments. We chose the attribute “sex” with values “male” and “female” as a sensitive attribute, as well as a combination of the attribute “sex” with the attribute “race” with values “white” and “others”, where we grouped together all other races, because all other races make up $$\approx 10\%$$ of the dataset. The dataset got preprocessed in such a way that our implemented kNN algorithm could work properly in order to have a better runtime.

Each dataset (synthetic and real) is split randomly such that 50% of the dataset serve as training data for model training, 35% for validation to determine emsembles, and 15% for testing the quality of predictions in the online phase.

To get a diverse set of classifiers, we train five different classifiers on our datasets: (i) Decision Tree, (ii) Logistic Regression, (iii) Softmax Regression, (iv) Linear Support Vector Machine, and (v) Nonlinear Support Vector Machine [12]. Given that we have two groups, this results in ten classifiers when we split before training, and five when training on the full dataset.

**Metrics.** Given that we aim for a good compromise of accuracy and fairness, we use metrics to assess the different algorithms in these two dimensions. We use the well known accuracy-metric commonly used to evaluate machine learning techniques. For fairness, we distinguish between global and local fairness. To study global fairness, we use the “unfairness part” of the metric given by Eq. 2 (setting $\lambda=0$), to which we refer to as global bias (lower values are better).

To measure local bias, we define a local region bias metric, which we call *local region discrimination (LRD)*:

5 $$\text{LRD}=\frac{1}{|G|\cdot|D|}\sum\limits_{t_{i}\in D}\sum\limits_{g_{j}\in G}\left|\frac{1}{k}\sum\limits_{z_{l}\in R_{t_{i},g_{j}}}z_{l}-\frac{1}{k|G|}\sum\limits_{g_{m}\in G}\sum\limits_{z_{l}\in R_{t_{i},g_{m}}}z_{l}\right|$$

where $R_{t_{i},g_{j}}$ is the local data region of $t_{i}$ comprising the kNN of $t_{i}$ in group $g_{j}$. In this metric the probability of a positive predicted label of each group in the local region is measured against the average probability of a positive predicted label amongst all points in the local region. In this way, the metric reflects the average local fairness.

Table 4 depicts a brief overview over the different parameters used and evaluated in our experiments. Using the experimental setup described in this section, we now discuss results we obtained.

### Comparative evaluation in terms of accuracy and fairness

We first present results we obtained when using different algorithms on our synthetic dataset in terms of accuracy, global bias, and local bias. The metric used for the Decouple and FALCES algorithms in the following results is the $\hat{L}_{\text{mean}}$ metric described in Eq. 2.

**Fig. 5:**
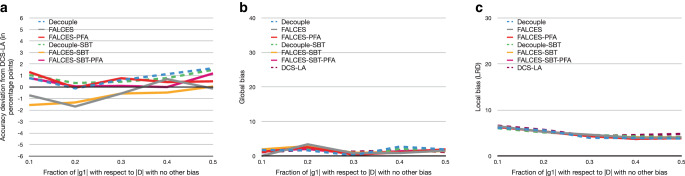
Results on synthetic data with varying group balance, no social bias, and no implicit bias using the $\hat{L}_{\text{mean}}$ metric. **a** Accuracy deviation from DCS-LA, **b** global bias, **c** local bias

As a first baseline, we start with a “clean” dataset with no social or implicit bias, and see if changes in group balance have an impact on our three metrics. Essentially, we expect only a marginal effect on accuracy and a low global and local bias, because the input data are a priori unbiased. This is confirmed by the results depicted in Fig. 5. Note that instead of plotting absolute accuracy for all methods, we plot the deviation algorithms have in accuracy from the accuracy reached by DCS-LA, reported as percentage points. DCS-LA is not considering bias and optimizes solely for accuracy, which is between 0.76 and 0.79 for DCS-LA over the whole range of considered group balance. The ordinate reporting percentage points, a deviation of -1 means that an algorithm reaches for instance 0.77 instead of 0.78 reached by DCS-LA.

In Fig. 5, we observe that all algorithms perform similarly, i.e., for all algorithms, there is some very small fluctuation in accuracy and global bias remains low. For local bias, while being generally low as well, we observe that it steadily increases with increasing imbalance, reaching a relative increase of up to 64% from the balanced case (0.5) to the highest imbalance (at 0.1, where only 10% of the dataset concern one group).

**Fig. 6:**
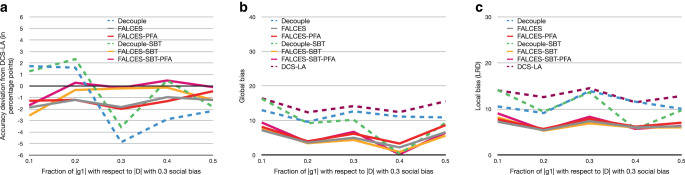
Results on synthetic data with varying group balance, 0.3 social bias, and no implicit bias using the $\hat{L}_{\text{mean}}$ metric. **a** Accuracy deviation from DCS-LA, **b** global bias, **c** local bias

Next, we perform the same analysis again, but this time with an additional social bias of 0.3 introduced to $g_{1}$. The results are summarized in Fig. 6. With the introduction of social bias, we observe that deviations in accuracy become more pronounced, in particular for the two variants of the Decouple algorithm. Least affected in terms of accuracy is FALCESSBTPFA, actually having comparable or better accuracy than DCS-LA. For both local and global bias, we see that all FALCES variants consistently outperform both Decouple variants and DCS-LA. Also, compared to the previous experiment without social bias, the field has overall shifted upwards. This shows that we cannot fully counter bias originally present in the dataset, but FALCES is best in reducing it while maintaining high accuracy.

**Fig. 7:**
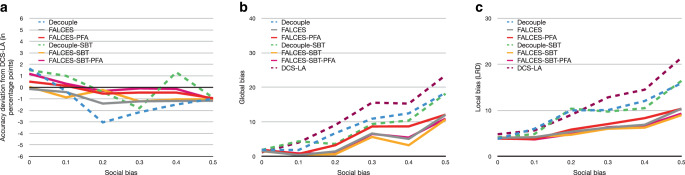
Results on synthetic data with varying social bias, group balance of 0.5, and no implicit bias using the $\hat{L}_{\text{mean}}$ metric. **a** Accuracy deviation from DCS-LA, **b** global bias, **c** local bias

**Fig. 8:**
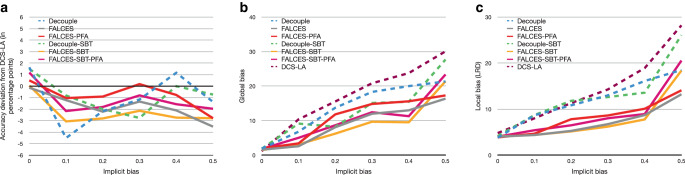
Results on synthetic data with varying implicit bias, group balance of 0.5, and no social bias using the $\hat{L}_{\text{mean}}$ metric. **a** Accuracy deviation from DCS-LA, **b** global bias, **c** local bias

Our next analysis focuses on the impact different degrees of social bias have on the overall performance, assuming balanced groups without additional implicit bias. Fig. 7 reports our results. For accuracy, we observe that all methods fluctuate, but the degradation in accuracy (typically less than 2 percentage points) is tolerable. Our approaches are more robust to social bias than the state-of-the-art Decouple variants, the PFA variants generally being closest to the accuracy reached by DCS-LA. For both local and global bias, a clear upward trend is visible with increasing social bias, showing that the more bias in the input data, the more bias the ensemble generates. However, the gradient of our approaches is less steep and consistently below the baseline methods. This means that the more social bias in the data, the more effective our approaches are in countering the bias to optimize (local) group fairness compared to the state-of-the-art. FALCES-SBT is best in terms of global and local bias, but FALCES-SBT-PFA has similar performance and thus presents a good compromise in datasets with mainly social bias.

We perform a similar analysis for implicit bias in the source data, again assuming a balance of groups (balance = 0.5) and setting social bias to 0. Fig. 8 visualizes the results of this set of experiments. Our first observation is that implicit bias impacts all metrics more than the previously considered social bias. As before, in terms of variations in accuracy, these are strongest for the Decouple variants, whereas the PFA variants of our algorithm outperform FALCES and FALCES-SBT. However, looking at both local and global bias, our algorithms without model pruning typically perform better than their counterpart with PFA. The reason for this is that model pruning during the offline phase can prune classifiers that would, during the online phase, be better compared to those retained after model pruning. Nevertheless, in general, our methods outperform the state of the art for a wide range of implicit bias configurations.

**Fig. 9:**
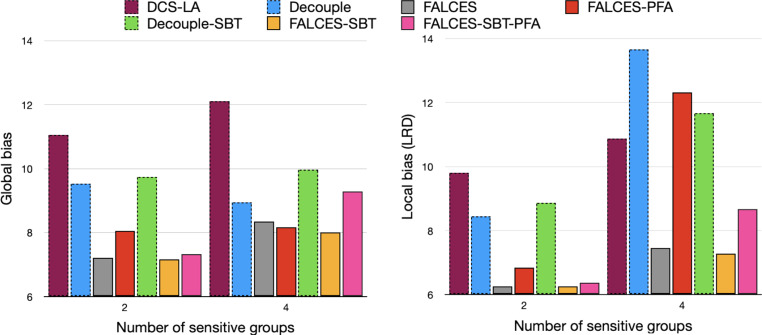
Global bias (*left*) and local bias (*right*) on real-world dataset for one or two sensitive attributes

We validate our findings on artificial data on the real-world dataset as well. Given that it includes two sensitive attributes (sex and race), we study accuracy, global bias, and local bias when just one attribute is used to form groups (resulting in two groups) and when two attributes are used (resulting in 4 groups). Fig. 9 shows the average results of multiple tests for global and local bias. Results on accuracy confirm that all algorithms perform similarly, it consistently ranges between 0.790 (Decouple) and 0.799 (DCS-LA). As before, we observe that FALCES variants typically are comparable or outperform the three baseline algorithms, both in terms of global and local fairness. With the increasing number of sensitive attributes, we observe that the bias increases for all methods.

In conclusion, we see that our methods improve on the state of the art by offering a better accuracy-fairness compromise than the state of the art Decouple approach (considering global fairness) and the difference in accuracy compared to DCS-LA is typically tolerable. Our methods are also the most robust to different types and degrees of bias (we studied group balance, social bias, and implicit bias). An added benefit is that our methods inherently consider local fairness as well, and our evaluation of local fairness shows that the classifications performed using our algorithms get us closer to equal opportunity for different predefined groups.

### Impact of different accuracy-fairness metrics

In Sect. 4.2, we have presented multiple alternative metrics for $af$, used both during model pruning in the offline phase and dynamic classifier selection in the online phase. All experiments so far have used our metric $\hat{L}_{\text{mean}}$ (Eq. 2). The next series of experiments investigates how different metrics potentially impact the result. For the sake of consistency and to compare the different metrics, we still use the same methodology (mean difference) to calculate the global bias and the LRD metric to calculate the local bias.

**Fig. 10:**
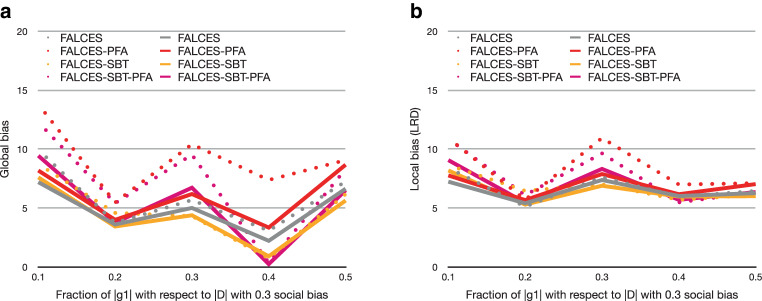
Results for varying group balance, 0.3 social bias, and using alternative $af$-metrics. The state-of-the-art metric results have dotted lines, while the $\hat{L}_{\text{mean}}$ results use solid lines. **a** Global bias, **b** local bias

First, we want to compare the results of the state-of-the-art metric of Eq. 1 as SOA, with our metric $\hat{L}_{\text{mean}}$, since it is an extended version of the SOA metric. As a reminder, our extension aims at countering the effect on fairness in presence of unbalanced groups. Therefore, we focus our study on evaluating both the global and local bias for different configurations of group balance. As before, accuracy is comparable across all approaches, whether we use SOA or $\hat{L}_{\text{mean}}$. Fig. 10 reports our results on global bias and local bias comparing the results of both approaches. For better readability, we omit the results of Deocuple, Decouple-SBT and DCS-LA, as their relative performance to the other approaches being analogous to our previous discussion. The dotted lines represent the results when using the state-of-the-art metric, while the results using the $\hat{L}_{\text{mean}}$ metric are presented in solid lines. For both global bias and local bias, we see that FALCES variants without model pruning (FALCES and FALCES-SBT) are comparable when using SOA or $\hat{L}_{\text{mean}}$. The effect of using a different metric only becomes apparent when model pruning is active. Overall, we see that $\hat{L}_{\text{mean}}$ closes the “bias gap” between FALCES variants with model pruning (FALCES-PFA and FALCES-SBT-PFA) and those without. This allows our methods to consistently exhibit low bias, especially in comparison to state-of-the-art algorithms like Decouple. This behavior can be explained by the fact the $af$ is used by model pruning where group imbalance can cause the pruning of otherwise good classifier combinations. Note that the use of $af$ during the online-phase is not sensitive to the choice of the two metrics, because it ensures class balance in the local region by selecting $k$ members of each group to form a region. Consequently, FALCES and FALCESSBT are not significantly affected by the choice of metric.

**Fig. 11:**
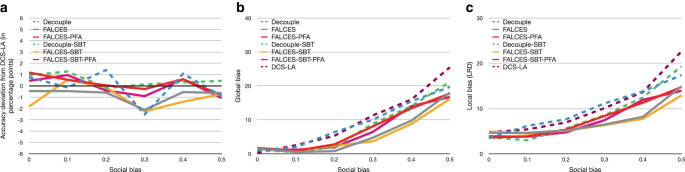
Results on synthetic data with varying social bias, group balance of 0.5, and no implicit bias using the $\hat{L}_{\text{impact}^{*}}$ metric. **a** Accuracy deviation from DCS-LA, **b** global bias, **c** local bias

**Fig. 12:**
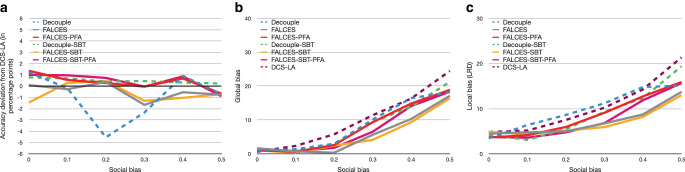
Results on synthetic data with varying social bias, group balance of 0.5, and no implicit bias using the $\hat{L}_{\text{elift}^{*}}$ metric. **a** Accuracy deviation from DCS-LA, **b** global bias, **c** local bias

Furthermore we want to explore the effects when using other fairness metrics, like $\hat{L}_{\text{impact}^{*}}$ or $\hat{L}_{\text{elift}^{*}}$ as described in Eq. 3 and Eq. 4 respectively. Due to the nature of ratio metrics in comparison to difference metrics, when using values within the range of $[0,1]$, the output generated by the ratio metric is greater than the output generated by the difference metric. Since the fairness part of $\hat{L}_{\text{impact}^{*}}$ and $\hat{L}_{\text{elift}^{*}}$ will generate values higher than the $\hat{L}_{\text{mean}}$ metric, we want to evaluate these metrics when varying the bias value of the dataset. The expectation is that a higher bias in the dataset results in a bigger improvement by our algorithm variants when using one of the two ratio metrics. Here we also want to measure the influence of these metrics on the accuracy value, since this is expected to decline. The results are depicted in Fig. 11 and Fig. 12. The results show, that although our FALCES algorithms still outperform the DCS-LA and Decouple algorithms, the gap of global and local bias is more narrow. Contrary to our expectations, the bias is not further decreased compared to the $\hat{L}_{\text{mean}}$ metric results. In general the global and local bias is even higher than when we used the $\hat{L}_{\text{mean}}$ metric. The difference between both ratio metrics $\hat{L}_{\text{impact}^{*}}$ and $\hat{L}_{\text{elift}^{*}}$ is very small as expected, since both metrics are similar to each other. Also the assumption about the influence on the overall accuracy is invalidated within this experiment. The overall accuracy values of the predictions are similar to the ones using the difference metric for the fairness part, hence metric $\hat{L}_{\text{mean}}$. However, the conclusion that the FALCES algorithms reduce further bias compared to the state-of-the-art Decouple approach still persists when using other $af$ metrics.

### Impact of different $\lambda$ values for the accuracy-fairness metrics

**Fig. 13:**
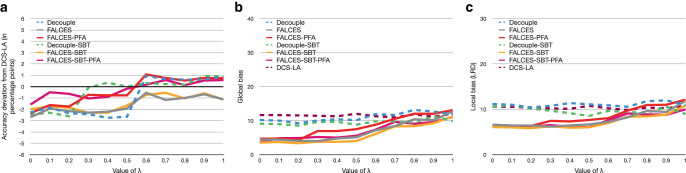
Results on synthetic data with 0.3 social bias, group balance of 0.5, and no implicit bias using the $\hat{L}_{\text{mean}}$ metric with a varying $\lambda$ value. **a** Accuracy deviation from DCS-LA, **b** global bias, **c** local bias

**Fig. 14:**
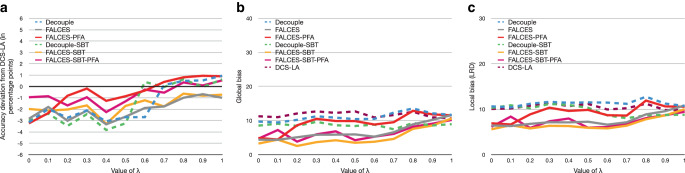
Results on synthetic data with 0.3 social bias, group balance of 0.5, and no implicit bias using the $\hat{L}_{\text{impact}^{*}}$ metric with a varying $\lambda$ value. **a** Accuracy deviation from DCS-LA, **b** global bias, **c** local bias

All experiments so far have used the default $\lambda$ value of $\lambda=0.5$, which means that both parts, accuracy and fairness, are valued the same. We want to test how the overall results change when different $\lambda$ values are used, ranging from $\lambda=0$ (ignoring the accuracy part) to $\lambda=1$ (ignoring the fairness part) with steps of 0.1. The evaluation has been made for the $\hat{L}_{\text{mean}}$ and $\hat{L}_{\text{impact}^{*}}$ metrics. We omit the evaluation for the $\hat{L}_{\text{elift}^{*}}$ metric as they are analogous to the results of the $\hat{L}_{\text{impact}^{*}}$ metric. Fig. 13 and Fig. 14 show the results.

The DCS-LA algorithm does not have the same result in each computation, which could be the result of randomly choosing the classifier if multiple classifiers perform equally well. Overall the results show, as expected, that for a low $\lambda$ value, the bias tends to be lower for our algorithms, but also worsen the overall accuracy. For a higher $\lambda$ value, the accuracy gets better, but the bias becomes higher at the same time. For the experiments we conducted, a $\lambda$ value between 0.3 and 0.5 seems to be optimal when using the $\hat{L}_{\text{mean}}$ metric, while for the $\hat{L}_{\text{impact}^{*}}$ metric a $\lambda$ value between 0.3 and 0.6 seem to provide similar results. Surprisingly, when using the $\hat{L}_{\text{impact}^{*}}$ metric, the FALCES-PFA algorithm seems to perform badly, while the FALCES-SBT-PFA algorithm overall performs the best considering local bias and accuracy, and also performs similar to the FALCES-SBT algorithm when considering global bias and accuracy. Also our FALCES algorithms have some different results compared to the DCS-LA algorithm, although it behaves similarily for $\lambda=1$. However, since we consider the kNN of each group nonetheless within FALCES, we take into account more sample points overall within the validation dataset compared to the DCS-LA algorithm.

### Impact of different $k$-values for the kNN algorithm

**Fig. 15:**
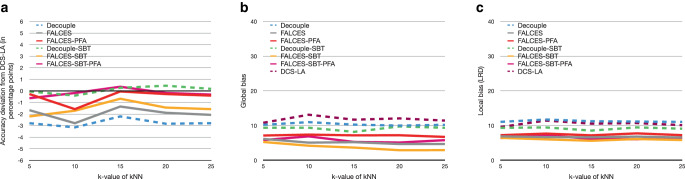
Results on synthetic data with 0.3 social bias, group balance of 0.5, and no implicit bias using the $\hat{L}_{\text{mean}}$ metric with a varying $k$-value for the kNN algorithm. **a** Accuracy deviation from DCS-LA, **b** global bias, **c** local bias

Another experiment we conduct in this paper is about the influences of different $k$-values for the kNN algorithms. A positive aspect of a high $k$-value is that multiple points are considered, hence the effect of a single point is lower. However this could also result in a problem, since if the $k$-value is too high, points might be considered as close which are not necessarily similar to the current point considered. Also we are taking into account the kNN of each group for the FALCES algorithms, so if the $k$-value is 20 and we have, e.g., 4 sensitive groups, we use 80 points to decide which model combination is best suited for the current point. The results are shown in Fig. 15.

The results of this experiment are unexpected as the accuracy results are the lowest for $k=10$. Apart from that the results seem to be quite stable, especially for the local bias value. The downside of having a small $k$-value is that not many points are considered in order to find the locally best machine combination for a specific sample. On the other hand, choosing a high $k$-value could risk that points are considered as being similar to the current tuple, although they might vary a lot. Especially when dealing with a lot of different groups, one group might only have very few samples. If a high $k$-value is chosen it would mean that a high proportion of this group is considered. E.g. we set $k=100$ and the underlying dataset consists of 6 different groups, whereas there is one small group consisting of only 300 tuples. In this case 1/3 of the group would be considered as being similar to the currently considered tuple, although in reality a high number of these tuples might be totally different.

### Impact of preprocessed data

**Fig. 16:**
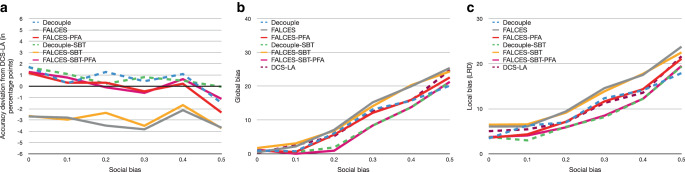
Results on non-preprocessed synthetic data with varying social bias, group balance of 0.5, and no implicit bias using the $\hat{L}_{\text{mean}}$ metric. **a** Accuracy deviation from DCS-LA, **b** global bias, **c** local bias

In Sect. 4.3 we mention that a preprocessed dataset is needed to perform the more runtime-efficient kNN algorithm. Additionally, it is also mentioned that not only the runtime might suffer if a no preprocessed data is used, but that also the qualitative results might take a hit. Therefore we conduct an experiment about the influence of using preprocessed data. In Fig. 16 we see the results of the experiment using the same data as the experiment in Fig. 7. The difference is that this time we do not use pre-processed values, hence we only have a distance of 1 or 0 of an attribute between two points. We use distance 0 if the attribute value is the same and 1 otherwise. The influence on the runtime is shown in Sect. 6.8. However, for the LRD metric in the evaluation we still use the original kNN algorithm.

As expected, the results are worse if the data is not preprocessed. However, the degree of the impact is higher than initially expected. Especially the variants without pruning model combinations perform worse in both aspects, accuracy and bias, compared to the Decouple, and even the DCS-LA algorithm. Also the FALCES variants with model pruning are providing similar results as the Decouple algorithms. This indicates that the FALCES algorithms should be used on preprocessed datasets.

### Evaluation of the Runtime-Efficient Framework Adaptation

**Fig. 17:**
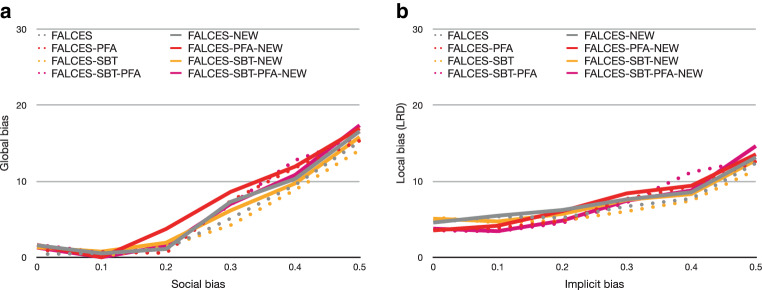
Results on synthetic data with varying social bias, group balance of 0.5, and no implicit bias using the $\hat{L}_{\text{mean}}$ metric. **a** Global bias, **b** local bias

The last experiment we conduct is comparing the algorithms implementing the new runtime-efficient framework adaptation described in Sect. 5 with the other FALCES algorithms. We chose $k=10$ for the kNN algorithm within the prediction dataset. The accuracy value is similar in both experiments and is therefore not depicted here. The results are depicted in Fig. 17. These were similar to the results when varying the implicit bias or using the other metrics. The FALCES algorithms using this new adaptation have added “‑NEW” to their name in this graph and are depicted as solid lines. The comparison of the runtime is provided in Sect. 6.8.

**Fig. 18:**
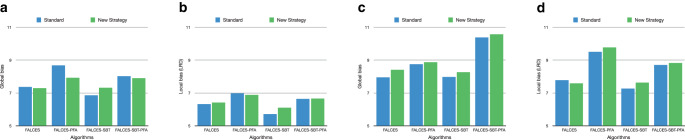
Results on the Adult Data Set using the $\hat{L}_{\text{mean}}$ metric with 2 sensitive groups (a & b) and 4 sensitive groups (c & d). **a** Global bias, **b** local bias **c** global bias, **d** local bias

Expectedly, the bias of the results slightly increase using this new approach. However the difference is mostly marginal. In situations where the runtime is important, it might be useful to use the runtime-efficient adaptation, since the tradeoff in terms of bias is rather low.

Furthermore we want to evaluate the runtime-efficient framework adaptation on the real-world dataset as well. Fig. 18 depicts the results in comparison to the standard FALCES algorithms for the dataset with both, 2 sensitive groups and 4 sensitive groups. The results of the standard FALCES algorithms slightly deviate from the ones shown in Fig. 9, since that figure shows the average of multiple runs. On the real-world dataset with 2 sensitive groups the FALCES algorithms using the framework adaptation even have less global bias for most of the variants. However, this can not be confirmed for the dataset with 4 sensitive groups. In general the bias value is very similar in both strategies.

### Runtime evaluation

**Fig. 19:**
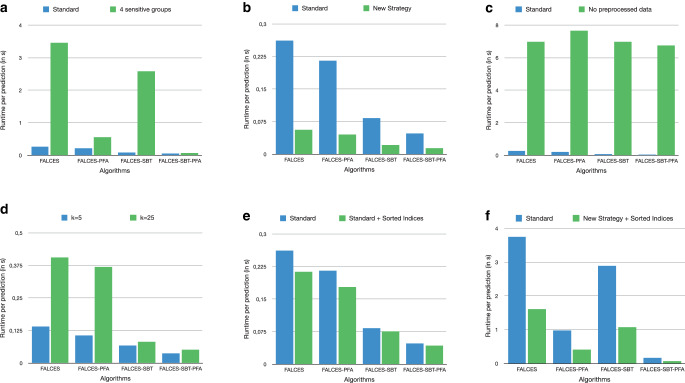
Runtime analysis. **a** Impact of the number of sensitive groups: 2 vs 4 groups, **b** standard vs New Strategy, **c** standard vs No Preprocessed Data, **d** impact of $k$-value on the runtime: 5 vs 25 kNN, **e** impact of using sorted indices: Standard vs Sorted Index Improvement, **f** standard vs New Strategy + Sorted Indices on the Adult Data Set with 4 sensitive groups

We also evaluate the efficiency of our approach in its online phase. In particular, we study the effect of model pruning in the offline phase on the online performance, as well as the effect of other proposed FALCES strategies. To this end, we run the four variants of FALCES and measure the average runtime to perform online classification on a tuple for which we want a prediction. Compared to our originally published paper [14] we provide a more extensive runtime evaluation in this enhanced version. Due to a different hardware, as well as some minor changes within the code, the overall runtime in this paper also deviates compared to the original paper. For Fig. 19a–e the evaluation has been conducted on the synthetic dataset with 30% of social bias, while for Fig. 19f the evaluation has been done on the real-world dataset.

We report results in Fig. 19a on our synthetic dataset with 30% social bias, on which we can vary the number of groups (either 2 or 4), given two sensitive attributes. In any configuration, we see that model pruning during the offline phase improves the average runtime to classify a test tuple during the online phase. While this improvement is moderate when limiting to two groups, the difference increases as the number of groups increases. This can be explained based on the fact that for two groups and five models trained per group, we have 25 combinations to consider during the online phase when none are previously pruned. This exponentially increases with the number of groups, e.g., for 4 groups, $5^{4}=625$ combinations need to be tested. The runtime gap is increasing with the numbers of model combinations considered. Surprisingly the SBT variants also outperform the other algorithms in terms of runtime by quite a margin. This could be due to a slightly different implementation strategy adapted for this variant.

The FALCES algorithms implementing the runtime-efficient framework adaptation defined in Sect. 5 show a big runtime improvement compared to the standard FALCES algorithms, as depicted in Fig. 19b. Taking into consideration the results of Fig. 17 that showed that the overall bias is only slightly increased for the framework adaptation with our settings, and in some cases even performs a little bit better, using this strategy seems very promising.

Fig. 19c depicts the runtime results of our standard algorithm compared to the runtime, if the data are not preprocessed properly and do not conform to a specific format. As expected the runtime increases significantly, since the optimized kNN algorithm cannot be used. Combining it with the results of Fig. 16 which showed that if the data are not preprocessed, FALCES does not reduce the bias compared to the state-of-the-art algorithms, which proves that preprocessed data are of utter importance for our FALCES algorithms, both in terms of runtime as well as quality of the results.

The $k$-value chosen has also an influence on the overall runtime as it can be seen in Fig. 19d. In this experiment, the runtime with 2 kNN compared to 5 kNN is tripled for the algorithms where the data are not split before the training phase. However, for the SBT variants, the runtime difference is surprisingly rather small. An explanation could be that due to splitting up the dataset beforehand, finding and accessing the kNN can be performed faster, while the amount of model combinations considered is still the same.

Fig. 19e depicts the impact of having sorted indices for the validation dataset. This shows that the runtime can be further improved using this strategy, while having no effect on the overall quality of the results.

Fig. 19f shows the effect on the runtime on the real-world dataset with 4 sensitive groups, when comparing the original FALCES algorithms with their runtime-efficient framework adaptations described in Sect. 5, combined with having sorted indices within the validation dataset.

### Summary

The results of the detailed experiment discussion can be summarized as follows.

- **Varying group balance:** The results of FALCES are quite stable over imbalanced datasets.
- **Different types and amount of bias:** Higher bias within training datasets results in higher bias of the results, although FALCES keeps the bias lower than state-of-the-art algorithms. In general the datasets containing social bias resulted in an overall lower bias of the results, than the datasets containing implicit bias. This means that social bias is easier to detect.
- **Metrics:** While using the adapted ratio metrics $\hat{L}_{\text{impact}^{*}}$ and $\hat{L}_{\text{elift}^{*}}$ also provide an improvement on the results, the gap to the state-of-the-art algorithms when choosing the mean difference metric $\hat{L}_{\text{mean}}$ is bigger and thus shows more promising results. A good $\lambda$-value depends on the metric used, but for the mean difference metric a value between 0.3 and 0.5 returned the best results.
- $k$**-value of kNN:** The results in our experiments are quite stable. Due to the increased runtime when choosing a high $k$-value, $k=15$ proved to be a good cutoff value.
- **Non-preprocessed (non-numerical) data:** While the results of FALCES are promising for most of the datasets, it has been shown that they are not viable to be used on non-numerical datasets without prior preprocessing. These were the only experiments in which state-of-the-art algorithms outperformed FALCES. Furthermore, the runtime on non-preprocessed non-numerical data is impractical.

While FALCES typically provided good results, which variant to choose depends on several factors.

- **Splitting the dataset (SBT):** No definitive answer to the question of whether or not to apply splitting can be given when focusing on the quality of the results, However, in most cases the results of the FALCES algorithms using this technique show a slightly smaller overall bias. Additionally, the runtime of the algorithms using this techniques is lower than the runtime of their counterparts.
- **Prune model combinations in the offline phase (PFA):** In general, pruning model combinations in the offline phase leads to higher bias in the results. The reason behind this is, that a model combination might perform badly when the whole dataset is considered, but it might be the most suitable model combination for a specific local region. However, not pruning model combinations might result in an unfeasible runtime. This heavily depends on the number of trained models and more so on the number of sensitive groups. If more than two sensitive groups are considered and/or the runtime is crucial, model pruning is needed. Furthermore, the results when pruning model combinations were a bit more accurate in general, but the improvement in accuracy did not outweigh the deficit coming from the increase in bias.
- **Runtime-efficient framework adaptation (‑NEW):** The main focus of the adaptation was to reduce the overall runtime, while trying to keep the quality of the results on a similar level. The results of the experiments show that it succeeded and that the adaptation variants are typically more practical.

Overall, FALCES-SBT-PFA-NEW is a promising algorithm, when the runtime is an important factor and FALCES-SBT or FALCES-SBT-NEW, if the goal is solely to reduce bias.

## Conclusion

This paper studied the novel problem of making locally fair and accurate classifications to foster equal opportunity decisions. We have presented two general frameworks to address the problem, as well as FALCES, an implementation of the frameworks that combines and extends techniques of dynamic model ensembles and fair model ensembles. Our experimental evaluation demonstrated that FALCES generally outperforms the state of the art when it comes to balancing accuracy and fairness for several types and degrees of bias present in the training dataset. The algorithm variant adaptations for the runtime-efficient framework show an improvement in terms of running time over their variants implementing the original framework, while having comparable bias values. Furthermore, multiple metrics to measure accuracy and fairness have been implemented and tested, but the results show that the biggest improvement of our FALCES algorithms over the state-of-the-art algorithms are achieved when using the mean difference metric for the fairness part, hence $\hat{L}_{\text{mean}}$. A good value for weighing the accuracy and fairness part is $\lambda=0.3-0.5$, but it varies a bit depending on the metric used. Although overall the FALCES algorithms are successful in improving the fairness of the results while still staying similarily accurate, it has been shown that not every data is suitable for our algorithms. Possible avenues for future research include methods that diversify the set of trained models in a controlled way or dynamic and adaptive setting of the parameter $k$ of the kNN search, depending on the density of the data region. While we already conducted some experiments in terms of choosing different $k$-values, these experiments can be further enhanced in the future to determine good $k$-values based on factors like dataset sizes and the number of samples per group.
